# Practical Medicine and Surgery

**Published:** 1849-11

**Authors:** T. S. Bell

**Affiliations:** Louisville, Kentucky


### WESTERN JOURNAL

OF

### MEDICINE AND SURGERY.

NOVEMBER, 1849.

### Art. I.—*Practical Medicine and Surgery.* By T. S. Bell, M. D., of Louisville, Kentucky.

The leading and important duty of medical literature is to present accurate pictures of disease, and approved methods of successful treatment. The day of attenuated theory and extravagant speculation is passing away, and more genial methods of investigation, than those formerly pursued, are rapidly displacing time-worn errors, and inducing salutary treatment of disease.

The duties of the successful practitioner of medicine are becoming much more onerous than they were under the old regime, because he has light now to guide him prosperously through the labyrinths of pathology. Under the ascendant stars of humoralism, chemical and mechanical pathology, the investigator was without compass, rudder or pole star, and the path to his haven was entirely of a conjectural character.

The genius of Bichat first opened the way to the healthful revolution that has taken place in pathological investigations. The zeal, energy, application and perseverance of that remarkable man conducted him to a noble eminence in medical science, and, although he was unable to entirely shake off the shackles of the predominant systems of his day, the world is indebted to him for the erection of that Pharos, which not only revealed the shoal waters of error, but shed its light upon the roadsteads of truth. The cultivation of Pathological Anatomy has made Practical Medicine almost a new science, and it never can command too much of the attention of the medical practitioner.

In a paper on the subject of cholera, prepared sometime since for this Journal, we took a hasty glance at the range of investigation that must necessarily be made by the practitioner for each prescription, and the importance of this subject is so great, that we shall make no apology for pressing it upon the attention of our readers again.—In Stille's *Elements of General Pathology*, a most excellent diagram is given of the matters of inquiry that must engage the attention of a practitioner in making a scientific prescription:

A. PRELIMINARY INQUIRIES.

| | | |
|---|---|---|
| *a.* | Age. | Profession, and social condition. |
| | Sex. | Habits of living. |
| | Temperament. | Diseases of Parents, Relations and Children. |
| | Constitution. | |
| | Idiosyncrasies. | Previous diseases. |
| *b.* | The probable or attributed cause of the illness. | |
| | The mode and period of the attack. | |
| | Premonitory symptoms present or absent. | |
| | The course of the disease. | |
| | The previous treatment. | |

B. THE PRESENT CONDITION OF THE PATIENT.

| | |
|---|---|
| *a.* The Exterior. | General appearance. |
| | Color of the skin and complexion. |
| | Expression. |
| | Degree of muscular development. |
| | Decubitis. |

- *b.* The Muscular System.
  - Strength.
  - Movements.
  - Paralysis.
  - Voice.
  - Convulsions.
  - Spasms.
- *c.* The Nervous System.
  - General and special sensibility.
    - Pain, its characters and intensity.
    - State of all the senses.
  - The Intellect.
    - Activity.
    - Delirium.
    - Illusions and hallucinations.
    - Coma.
  - Sleep.
    - Duration.
    - Dreams.
- *d.* The Digestive Apparatus.
  - Appetite.
    - Degree.
    - Perversions.
    - Thirst.
  - The Tongue.
    - Size and shape.
    - Color.
    - Coat.
    - Moisture.
  - Salivation, Mastication, Deglutition.
  - Abdomen.
    - Nausea
    - Vomiting.
    - Pain.
    - Sensibility on pressure.
    - Stools, { Their frequency, quality, &c.
    - Piles.
    - Matters vomited.
    - Size of abdomen.
    - Tumors.
    - Effusion.
    - Dimensions, &c., of liver, spleen, kidneys, and bladder.
- *e.* Respiratory Organs.
  - The signs revealed by
    - Inspection.
    - Mensuration.
    - Auscultation.
    - Percussion.
- *f.* Circulatory Organs.
  - The Heart. . . Physical examination.
  - The Arteries. . Pulse. Sounds.
  - The Veins. . . Development. Murmurs.
- *g.* The Skin.
  - Color.
  - Dryness.
  - Temperature.
  - Sensibility.
  - Eruptions.
- *h.* The Excretions.
  - Perspiration.
  - Sputa.
  - Urine.
  - Feces.
  - Menstrual fluid.
  - Inquire, where appropriate, into their
    - Quantity,
    - Color,
    - Peculiarities of form,
    - Consistence,
    - Specific gravity,
    - Mixture,
    - Odour,
    - Taste, and
    - Reaction with chemical tests.

These are the mere externals of inquiry; they are but the names of large fields of thought, that must be explored for the development of action. The pathological condition of the organs, of which the diagram gives only signs or symptoms, and the therapeutical means for changing the morbid actions into healthy ones must also pass in review before the mind of the practitioner. What a world of intelligence and judgment is required for that act, which is often performed as the merest adventure! The diagram we have given is an index to the labors of the physician who lives in harmony with his profession, and we have given it in order to awaken the careless, to the immense importance of the business of prescribing. He who falls below the standard laid down in this diagram, must often guess his way, and do evil, where good might have been accomplished.

These hints are but preparatory to a synopsis we design to present of the practical details of recent medical periodicals. We know of nothing we can lay before our readers of more interest and value than a record of the efforts of practising physicians to relieve suffering. In this way the experience of the many becomes the property of each individual, and the rays of medical light, scattered over an immense field of vision, are gathered to a focal point, where all may feel their enlightening influences.

*Surgery.*—The August number of the Dublin Quarterly Journal of Medical Science contains an interesting and valuable paper on Cervical Abscesses, by William Hargrave, Professor of Surgery in the Royal College of Surgeons in Ireland. The observations of Dr. Hargrave are upon subjects that frequently occupy the attention of the physician, and it is necessary that he shall know the perilous character of those operations which may end in death, when he least expects such a result. We quote three of these cases in the hands of Dr. Hargrave:

**1.** "An abscess formed on the right side of the neck in a young child; a peculiar thrill and tremor was observed in it; after much deliberation it was punctured, pus mixed with blood flowed from it, then pure blood; the orifice was closed: the child died that evening. The post mortem examination exhibited a cribriform communication between the abscess and the external jugular vein."

2. Mr. Liston punctured an abscess on the right side of the neck, with which the common carotid artery had communication. The artery was tied low down in the neck. On the fifteenth day the patient died, and it was found that the artery had opened into the abscess.

3. An abscess formed spontaneously in the neck five months before death ; a white purulent discharge from a fistulous opening in the supra-sternal fossa remained, from which arterial hemorrhage occurred ; it was arrested by compression, but returned on this means being discontinued. The patient sank in forty-eight hours from the first invasion of the hemorrhage. The post mortem examination exhibited an old abscess of a large size, occupying the front of the neck below the larynx, and extend ing so as to reach the arch of the aorta, and the arteria innominata. The cellular coat of the arteries was removed, and the fibrous one to some extent ; and a lacerated opening of the arteries, about a quarter of an inch in length, penetrated the lining membrane.

There are few who feel the perilousness of hemorrhage in opening abscesses of the tonsils, but Dr. Hargrave records dangerous cases of this operation. Two sources of danger are found in the tonsillitic arterial circle, and the ascending pharyngeal artery. These, though perilous, need not be fatal, but the third source of danger must be instantaneously fatal. This fatality is from wounding the internal carotid artery.

These are matters of fact that cannot be too vividly impressed upon the mind of the practitioner. We have known of fatal cases of hemorrhage from ulceration about the tonsil, and the imminent danger of ulcerative process in that region, must be apparent to any one who has before his mental vision the arterial ramifications in the region about the tonsils. And when suppurative action, as a sequelæ to scarlatina or strumous inflammation, has taken place in the tonsils, it is well enough for the practitioner to remember that incisions into the sac of the abscess may reveal to him a scene of the most urgent danger. Let it be granted that this danger is a rare event; this rarity of itself throws over the case the necessity of caution, and shows the value of the admonitions, which Dr. Hargrave has thought proper to urge upon the attention of his medical brethren. He says: "So critical and serious are these consequences in practice as to demand every circumspection and examination before abscesses in this region are punctured, but also active subsequent watching in order to detect the first symptom of bleeding, whether of the more evident or of the doubtful kind. We should, therefore, be prepared to meet and ward off this unpleasant, if not fatal occurrence, in cases that otherwise promise a most satisfactory termination. From an attentive examination of the abscesses which have been complicated with active losses of blood, I find that the majority of them were in subjects of a more or less cachectic habit, suffering from struma, or bad forms of scarlatina, perhaps also of a cancerous diathesis." We cannot too strongly urge upon the attention of our readers these valuable and important admonitions. They should not be neglected by any one who has anything to do with cervical abscesses.

In illustration of the dangerous character of these abscesses, we must condense an interesting case reported by Dr. Hargrave, and we do it more to show the treatment, than to impress the lessons taught by Dr. Hargrave as to the character of cervical abscesses.

In January, 1849, a patient, aged sixty-one, was admitted into the City of Dublin Hospital. He had a tumor occupying the space between the symphysis and the angle of the jaw on the left side. The tumor was firm, but careful examination showed fluctuation. When punctured, a quantity of straw-colored serum, mixed with scrofulous-looking matter was discharged. The abscess was then poulticed. On the following day, the patient was seized with rigors and an intense headache, sickness of stomach, and pain in the tumor. These symptoms were followed by erysipelas. Under appropriate treatment the discharge from the abscess continued to improve up to the 20th of January, seven days after the abscess was opened, something like grumous blood appeared on the poultice. Three days after, in the night, a gush of blood came from the cavity of the abscess; it flowed per saltum —was of a florid color, and was arrested by placing the finger on the wound. When this pressure was taken off, the blood came out, in a jet the thickness of the little finger, and was thrown fully a yard distant. A consultation determined upon tying the common carotid, and the operation was performed by a transverse incision made two fingers' breadth above the clavicle; the sterno-mastoid muscle was then carefully divided its entire breadth upon a director, and the vessel was then easily secured. As soon as the ligature was drawn upon the vessel, the hemorrhage was at once arrested. Under appropriate treatment the case went on favorably until the 5th of February, when another hemorrhage occurred, which prostrated the patient very much. The wound was dressed with matico leaves moistened with hot water, laid thickly over the incision; over these a dossil of lint was placed, well saturated with an infusion of the leaf, and secured by adhesive plaster. The wound was daily dressed in this way. In twenty days and a half the ligature came away, and there was no more hemorrhage.

Dr. Hargrave is strongly impressed with the belief that the *matico* dressings were of material benefit in restraining the hemorrhage, and he testifies to its value as a styptic in other cases that have come under his observation.

We have given the principal features of this paper on Cervical Abscesses, because the facts are important and should be generally known. And they are the more important because these cases usually fall into the hands of general practitioners, who are not prepared to perform such surgical operations as are necessary in taking up the subclavian or common carotid.

Dr. Hargrave lays a great deal of stress upon his novel mode of reaching the carotid by a transverse, instead of a longitudinal incision. Dr Hargrave says: "From the facility of securing the artery by the plan now proposed, I consider it deserving of a place in the systematic works in this department of Surgery, and would term it the *transverse operation, or section of the sterno-mastoid muscle for the ligature of the common carotid artery.*"

We cannot quit this department of the subject, without calling attention to the merits of the *Matico*, referred to by Dr. Hargrave as a valuable styptic. A great deal of testimony has accumulated now, on the character of the matico as an agent for controlling hemorrhages, and, as it has not found a place in the systematic treatises on materia medica, we shall supply the deficiency by bringing forward the history of the use of the Matico.

In 1839, at a meeting of the Provincial Medical and Surgical Association of York, Dr. Jeffreys of Liverpool, introduced the Matico, as a styptic. The botanical name of the plant is Piper Angustifolium, and it is a native of Peru. Soon after Dr. Jeffreys called attention to it, the Matico, as an external application, was used with success in the Dundee Infirmary, by Dr. Munro. It exerted a happy influence over a hemorrhage from "a considerable branch of the temporal artery." Compression and cold applications failed to give any relief, but the application of the leaf, *not the powder*, arrested the hemorrhage. A hemorrhage from a wound of a branch of the palmar artery was treated in the same way with equal success.

The external use of the leaf having proved highly suc cessful, a resort was made to its internal use. Dr. Jeffreys reports the case of a patient "who had been subject for two months to excessive discharge of pure blood and coagula from the vagina, amounting to nearly a quart in a few days, occurring every ten days or a fortnight, and followed by a serous and muco-purulent discharge." The usual treatment failed, and health was restored in a few days, by the use of a wineglass full of the infusion of Matico four times daily. In another case a hemorrhage from the bowels, an infusion of Matico, in the proportion of half an ounce to the pint, of which three tablespoonfuls were taken every four or six hours, cured the patient by the use of three doses.

In hemorrhage of the bowels, during fever, Dr. Watmough states, in the Provincial Medical Journal, that the infusion of senna leaves and the matico, two drachms of each in a pint of boiling water, and taken frequently in doses of a wineglass measure, is very successful, particularly in the melæna of typhus fever.

Dr. Horne, of London, thus speaks of Matico, as an anti-hemorrhagic medicine: "A patient suffered from an alarming epistaxis, occurring spontaneously, in October last. All remedies of the usual character failed; the case was considered beyond the reach of relief, and the brothers of the patient were sent for. One of the brothers commenced the use of the Matico, and in six hours the hemorrhage ceased, after having continued for days.—There is a hemorrhagic diathesis in the family, and it has descended to the children of one of the brothers."

A relative of the family was rapidly sinking towards the grave, from disease and uterine hemorrhage, the latter of which baffled the ordinary remedies. Under the use of the Matico, the bleeding ceased, and the patient recovered.

These are the testimonials to the value of Matico, as a styptic, which we have gleaned from the European medical journals. We are far from supposing that this Peruvian plant will displace all other means for arresting hemorrhage; the utmost that we hope for it is, that it is really useful, and may take its place confidently among the means for controlling dangerous bleeding. It is due to the time-honored standards among the anti-hemorrhagic remedies, to say they have never yet failed in our hands.

*Arsenical preparations.* The French evidently pay but little attention to the progress of medicine in England.—The use of arsenical preparations in periodical diseases has recently been recommended in one of the leading medical journals of Paris, as something novel, and the testimony that usually dances attendance upon all *new* remedies, is gravely brought forward to favor arsenical preparations to the exclusion of Peruvian Bark. In the grave forms of fever and ague, there is no medicine that is comparable to Quinine. In an experience of nearly eighteen years, in a region where all forms of periodical disease may be seen, quinine has never disappointed us but once, and when given properly it excels all other remedies. In order to derive full advantage from its use, it should be given at least eighteen or twenty hours before the accession of the chill. As a general rule it is best to give the full dose at once, say 20 or 25 grains. If these rules are attended to, the failure to interrupt a chill will be a rare event. We have personally used broken doses, and the whole dose at once, and the latter is infinitely preferable. It is less troublesome to the patient, and his friends, secures rest and freedom from annoyance, and will more certainly remove the disease. This is not only the result of personal experience, but also of a great deal of observation. We have found, however, that females do not tolerate the full dose as well as men do. In the former, it creates more disturbance in the head, than the divided doses.

We should regret to see the arsenical preparations introduced generally into practice. They require a great deal of watching, much more indeed than is given to cases of chill and fever, and without vigilance, the remedy must do a great deal of harm. There must not only be an entire absence of all inflammatory action, but all tendency to it must be absent in order to tolerate the arsenical preparations. There are few cases, indeed, of fever and ague that will not yield as readily to other medicines as to the arsenic, and there can be no apology for its use in cases that will do as well with less dangerous articles. The arsenical preparations require a great deal of close watching, and they must be discontinued as soon as they manifest their peculiar action on the conjunctiva, or the stomach.

We have quoted in another part of the present number of this Journal, some remarks on the use of arsenic in certain affectious of the skin. In those cutaneous diseases that have a furfuraceous character, especially some forms of porrigo, Fowler's solution is an invaluable remedy. Dr. Hunt, of England, has called the attention of the profession, very forcibly to the use of this solution in all diseases of the skin that have the character we have mentioned. There are forms of erysipelas in which furfuraceous signs show themselves, and they will be materially benefitted by Fowler's solution. This, we regard as greatly the safest, best and most manageable form of arsenical medicine. But the practitioner must not forget that arsenical preparations are inadmissible in any cutaneous affection attended with active inflammation. The class of skin diseases in which arsenic is admissible is a small one. The experience of those who have used it most says: that among the exanthemata, *urticari tuberosa* is the only one that can be benefitted by arsenic. In papular diseases, *lichen* is the only one that can tolerate arsenic, and it can do so, only in cases when the chronic character is well marked. Among the vesiculæ, *chronic eczema*, when assuming the squamous character, is the only one that can improve under the use of arsenic. In pustular diseases, arsenic is not advisable, but it has been used successfully in *impetigo*, under the restrictions we have endeavored to enforce. But it is in squamous affections of the skin, when they become chronic, and especially in *lepra* and *psoriasis* that the arsenical solution most triumphantly acquits itself.

We cannot enforce too strongly upon the memory of our readers the necessity of caution in the use of arsenic. A remedy of such power requires great judgment, and the physician who uses it cannot be too cautious in prescribing it, nor too vigilant in watching its effects upon the system. As soon as any untoward symptom shows itself, the arsenic must at once be discontinued. The judicious use of this agent will not disappoint the vigilant physician, but the negligent one should by all means let it alone.

*Neuralgic affections.* This form of disease occasionally baffles all ordinary attempts at relief. We have seen cases in which all the agents of the materia medica were set at defiance, and which raged uncontrolled under the use of purgatives, antispasmodics, tonics, and powerful anodynes. A case of this kind is vividly impressed on our memory, and in order to give some assistence to those who may be bewildered as we were, we subjoin a history of the leading features of the attack.

Mrs. H. S. was delivered in 1845 of her first child.—The labor was not attended with any unusual occurrence, and the mother passed the first two days free from suffering of any description. We have never seen a case that was more propitious in all its characteristics, up to the beginning of the second week. At that time the patient complained of pain in the right groin, extending down the thigh, towards the knee. We feared, at first, that phlegmasia dolens was about to show itself, but all the diagnostic symptoms of that disease were absent, and the case soon showed the unmistakable signs of neuralgia of a very grave character. The pain was of a most excruciating character; the patient could not tolerate the least motion of the limb. There was no constitutional disturbance; all of the system, except the leg, was in a healthful condition. The lochial discharge was as it should be, the bowels regular, and the appetite good.

The treatment consisted of local applications, with a great variety of internal remedies. The most powerful stimulating liniments that could be borne were freely used; the limb was enveloped in hot fomentations; and all measures proved futile. The various preparations of opium, and other anodynes, such as lactucarium, conium, henbane, &c., failed to give any kind of relief. During the fortnight that these remedies were used, there was no mitigation of the suffering. The iodide of potash, the vinous tincture of colchicum, and all other remedies of this class were tried freely and faithfully without any useful result. The patient was nearly worn out with suffering and loss of sleep, and the case assumed a desperate appearance. In this state of things, the continued affliction of the sufferer, and the failure of all common means for her relief, we remembered a suggestion of Johnson's Materia Medica, that a camphor vapor bath often proved powerfully anodyne. The suggestion was immediately improved, by placing the patient in a cane-bottomed chair and disposing quilts around her so as to confine the vapor properly. An ordinary tin pan was placed over a spirit lamp resting on the floor, under the chair, and as soon as the pan was thoroughly heated, half an ounce of powdered camphor was thrown upon it. A powerful vapor was thus created, and so perfect was its effect that the patient could scarcely credit her own sensations when she found herself free from pain. This was her first respite for nearly three weeks. She enjoyed a refreshing night's rest, and next day seemed entirely well. On the following day she was much alarmed by the commencement of pain in the left groin, and for some hours the suffering was severe. In this case the pain extended upwards, instead of downwards, as in the first attack. It occupied one half of the abdomen, the linea alba being the dividing line between the healthy and the suffering parts of the abdomen. The patient, a very intelligent lady, declared that the bowels on the left side of the line were in the most intense agony that could be conceived of, while the portion of the intestines on the right side was free from any kind of uneasiness. The camphor vapor bath was again resorted to, and perfectly relieved the sufferer. In a subsequent labor, this patient recovered without any return of this singular and obstinate neuralgia.

We have often used this remedy since it was resorted to in this case, and in no instance has it ever disappointed our expectations, and we commend it to the attention of our professional brethren as well worthy of use.

*Croup.* Dr. Horace Green of New York has recently published a work "On the Pathology of Croup, with remarks on its treatment by topical medications," which should command the confidence of the profession. In this new work, Dr. Green enforces with ability, the novel treatment he had previously recommended for laryngeal inflammations, in a treatise on "The Diseases of the Air Passages." The new work on croup has received a very flattering reception in England. The British and Foreign Review speaks of it in terms of warm commendation, and the Dublin Quarterly Journal of Medical Science, with some little disparagement, speaks favorably of the book.

The fundamental principles of Dr. Green's pathology of croup, are that it is essentially an "inflammation of the secreting surfaces of the fauces, larynx and trachea, which is always productive of a membranaceous or an albuminous exudation," and that this "invariably commences in the superior portion of the respiratory passages, and extends from above downwards,—never in the opposite direction."

We shall not pause to call in question the universality of the truth of these propositions. Our particular object is to direct attention to Dr. Green's novel treatment. He recommends a solution of the crystals of nitrate of silver, from two to four scruples to the ounce of distilled water, as the topical application. The instrument with which this is to be applied is "a slender piece of whalebone, about ten inches long, slightly curved at one end, to which curved extremity is securely attached a small round piece of soft sponge, with a diameter of not more than half an inch." We would suggest the propriety of a very thorough examination of the sponge, to see that it is free from sand and grit.

Dr. Green says:

"The instrument being prepared, by suitably saturating the sponge with the solution to be applied, and the head of the child being firmly held by an assistant, and the base of the tongue depressed with a spoon, or any other suitable instrument, the operator carries the wet sponge quickly over the top of the epiglottis, and on the laryngeal face of this cartilage; then passing it suddenly downwards and forwards, passes it through the opening of the glottis into the laryngeal cavity."

There may be a fear, among those inexperienced in this topical application, arising from the strength of this solution. The fear is, however, groundless. The mucous membrane bears this solution remarkably well. Some time ago, Mr. Guthrie, the English surgeon, denied very indignantly that he had recommended an injection of a solution of nitrate of silver, twelve grains to the ounce of water, in gonorrhea, and utterly condemned the practice. But we have repeatedly seen a solution of twenty grains of nitrate of silver to the ounce of water, used in the early stages of gonorrhea, with great benefit to the patient.

Upon another point Dr. Green thus speaks:

"Ordinarily, I have applied in croup, a solution composed of from two scruples to a drachm of the salt, dissolved in one ounce of distilled water. A remedy of this strength I have freely applied to the fauces, pharynx, and into the larynx of young children, in a large number of cases during the last eight years, and in no single instance have I observed any indications of the dangers of suffocation from its employment. On the contrary, I have repeatedly observed, and have once before remarked, that much less bronchial irritation is produced by the application of the nitrate of silver into the larynges of young children who are suffering from croup, than where it is introduced into those of adults who are affected by chronic disease of the larynx."

The views of Dr. Green, though novel, are perfectly rational, and deserve confidence.

*Chloroform in Cases of Parturition.* When etherization was first announced, the opinion was confidently entertained that an innocent and salutary anesthesia might be induced whenever circumstances demanded it. The application of ether in dental operations was hailed with great satisfaction, and its speedy extension to the highest departments of surgical art naturally followed. The most confident assurances were given that ether was applicable to all cases, and that no one need suffer pain who wished to avoid it. In the midst of these cheering accounts, contradictory reports were given, and both serious and mortal cases were recorded as the direct result of etherization. The latter features of etherization increased so fearfully that its advocates diminished rapidly, and there was reason to suppose that the attempt to resort to anesthesia universally, would have the effect of driving etherization into total disuse. In the midst of these expectations, a new anesthetic agent was announced, and Dr. Simpson, who introduced it into midwifery practice, indulged strong hopes, that what had been labor heretofore, was to be a pleasant dream of Paradise. The most confident statements were given to show its harmlessness, and to beat down all opposition by strength of assertion. Dr. Simpson has shown the zeal of an apostle in his efforts to induce the use of chloroform in cases of labor, and evidently hopes that he has created a new branch of art, which he calls "Anesthetic Midwifery." In a recent pamphlet, published by Dr. Simpson, we are told that the author had administered chloroform in 245 cases of labor, and of this number five died, a statement which conclusively shows that chloroform has no tendency to dimimish mortality in parturient cases, for the loss of one patient in forty-nine, is rather unusual, even in Evrope. A full examination of the documentary testimony that has accumulated on this subject, goes to show that the sanguine expectations formed of the superlative good of anesthetic agents in midwifery are far from a chance of being realized.

We purpose, in these remarks, to make something of a survey of the use of chloroform, and to ascertain its positive merits. To Dr. Simpson, of Edinburgh, the profession is indebted for the introduction of this anesthetic agent, and he has been diligent and zealous in his efforts to urge its merits upon his professional brethren. Anesthetic medicine is, comparatively, a new field, in which there is yet much to observe, and in which much improvement may be made. We have not yet years of observation, in matters of anesthesia, on which to found positive principles of practice, and every thing that contributes to throw light on the interesting questions of anesthetic medicine should be welcomed with sincere satisfaction.—It is a fearful thing to use an agent capable of destroying the powers of the medulla oblongata, and thereby destroying life. An occasional occurrence of this kind is sufficient to make us pause, and deliberate well before yielding to the fascinations of the theory of anesthesia. At the same time, we cannot be indifferent to its demands upon the practitioner of medicine. Between the enthusiastic admiration of zealots, and the cold calculations of the timid, there is a *juste milieu* in which duty and prudence may meet.

From a careful analysis of the experiments and observations that have been made by those qualified for the interesting duty, we gather the following physiological results : "Chloroform requires about 288 parts of serum of the blood to dissolve it ; and taking the calculation that the body contains, on an average, about twenty-six pounds of serum, twenty-four minims is about the twenty-eighth part of the quantity the blood will take up—the quantity, consequently, for producing complete insensibility." Dr. Snow believes that anesthetic agents "produce their effect by their mere presence, impeding those combinations between the oxygen in the arterial blood, and the nervous tissues, on which the functions of the nervous system depend."[1] If these views are correct, and we see no reason to doubt the fact, we can be at no loss to account for the fatal issues that have been recorded in anesthetic medicine. When we impede the proper actions of arterialization and of the nervous tissues, on which life depends, we are unable to say to this agent thus far, and no farther shalt thou go. It is evident that fatality does not always depend upon mere idiosyncracy, nor upon diseases of the heart, lungs or arteries, which may be easily detected in the latter class, but upon an unseen and inscrutable condition of the nervous system itself. We cannot always reason ourselves into safety in the administration of anesthetic agents, and nothing could be much more deplorable than the consciousness that in an effort to destroy pain, we had taken life itself.

Mr. Gruby, by experiments of the Magendie order, finds the following results ;

"The arterial blood is redder (or at least as red) where chloroform has been inhaled, than (or as) where it has not."

"The venous blood becomes of a clear red color under the use of chloroform, losing its usual reddish-black tint."

"Venous blood in an animal under the influence of chloroform is redder than non-chloroformized arterial blood, and nearly as scarlet as such blood when penetrated by chloroform."

Here, then, is an unnatural state of things in the vital fluids of the body, which is sufficient in itself to awaken inquiry, and which admonishes loudly to prudence. The administration of agents capable of exerting an immense control over the sanguineous and nervous systems, and which cannot themselves be controlled, is no light or trivial matter. The action of the surgeon who undertakes to perform an operation which he thinks may destroy life, but who knows that the diseased condition demanding the operation will perform the same melancholy office, is altogether a different matter from the use of anesthetic agents, which may destroy life, without tending in any degree to save it. The mere existence of pain does not destroy life, and anesthetic medicines do not pretend to do anything more than the mere obliteration of consciousness.

When death first showed itself in the flowery paths of anesthesia, the zealous advocates of chloroform and ether endeavored to account for the deplorable result by the most foolish and garrulous reasoning. Logic in swaddling clothes always looks pitiable and miserable enough, but never more so than when it endeavors to palliate blunders that have taken life away. Mr. Meggison, a surgeon, of Newcastle, England, met, in his own practice, with the first fatal case of chloroformization, and, as an honest man, and one who feels the imperative responsibility of the medical profession, he came before the public with all the details of the case. He did not seek to blink the question, but honestly confessed that his patient died by the use of chloroform. Mr. Simpson, of Edinburgh, at once reviewed the case, and gave a specimen of special pleading in behalf of his hobby that would do no discredit to the renown of that firm which is immortalized in the "Pleader's Guide." In obedience to his mission, he distorted the plain statements of Mr. Meggison, and under the combined influence of perversion and a neglect of the facts, Mr. Simpson easily persuaded himself that chloroform had nothing to do with the death of Mr. Meggison's case—a conclusion that few could reach after reading the detail given by Mr. Meggison. There has been a fearful accumulation of death under the influence of chloroform since Mr. Meggison's case, and the impressive lessons given by this fatality have not been without their influence. That indiscriminate use of chloroform which threatened to inundate the civilized world has departed; routine and indiscreet practitioners have become timid, and the public have learned to be cautious. There are enough *recorded* cases of fatal and serious results in the use of anesthetic agents to admonish men of the fearful powers of these compounds, and we know enough to enable us to say understandingly that a large number of the fatal cases have not been reported.

Upon these premises it becomes a question of some interest to determine whether a practitioner should resort to the dangerous use of anesthetic agents, for the mere purpose of destroying the consciousness of pain, during the brief time employed in a surgical operation, or in the more protracted suffering attendant upon parturition. If the question were put to the practitioner whether chloroform should be used in any case where there was reason to apprehend death, a negative answer would be responded promptly. What, then, should it be in the circumstances universally acknowledged, that we have no means of determining a priori whether resuscitation or death will be the result of the administration of chloroform?—The fact that it has been taken once with impunity does not guarantee a similar result in a second trial. The fatal case which occurred a short time since in the New York Hospital is proof of this, for the patient had previously taken chloroform, and recovered from the effects without any trouble, but in the second trial he died.

While the zealous advocates of chloroform looked upon the first cases of fatality, they were for awhile astounded, but, determined to hang on to their hobby despite of light and knowledge, they rushed into the delusion that fatality was the result, not of the chloroform, but of the mode of its administration. They roundly asserted that death came from the exclusion of atmospheric air, while inhaling the chloroform, and confidently affirmed that the life of the patient, and, what was equally as dear to some of these gentlemen, the use of anesthetic agents, might be saved by a proper method of inhaling the chloroform. But, alas for the cause, it was found that fatality still moved attendant upon the improved inhalation, and the reluctant verdict was admitted, that death was the result of the chloroform *per se.* Of the fatal effects in many cases of chloroformizing, and of the serious consequences in numerous instances, there can be no kind of doubt. And in view of this state of things, what are we to think of the statements and logic contained in the following remarks by Dr. Bigelow: "if there is one consideration calculated to arrest attention in the history of etherization, it is, that although the anesthetic agents have been open to liberal use in every part of the civilized world, whether ignorantly, experimentally, or carelessly, although thousands have experienced their good effects, and although the physiologist, the ether opponents, and the coroner, have been equally ready to seize upon, and to exaggerate any case of accident that might seem to fall within their range—yet it is probable that the number of cases thus publicly suspected, have been less than ten, while the conclusive instances of direct relation between the anesthetic agent and death, are two in number. Can antimony or opium show as clean a bill of health for the same period? Can leeches or the lancet do better?" This is certainly an extraordinary statement, and the more so from being endorsed by the American Medical Society, by its publication in their Transactions. It is humiliating to see such a gentleman as Dr. Bigelow, a shining light in a noble profession, offering such blind incense to an idol. At the very time that Dr. Bigelow was offering this testimony to the comparative harmlessness of etherization, medical men were abandoning it on account of its fatality, and were blindly rushing to another anesthetic, under the view halloo of Dr. Simpson. Rabelais has immortalized such things in his record of the sheep of Panurge, which jumped over the precipice into the sea, because the bell-wether tumbled over. The abandonment of ether for another agent of anesthesia, stamps the character of Dr. Bigelow's statement of the harmlessness of etherization, and the apologies, the twistings and turnings of the chloroform-bitten advocates, for its fatal issues, speaks of the substitute in language not to be misapprehended.

The logic of Dr. Bigelow is exceedingly objectionable. In the hands of the discreet, intelligent physician, antimony, opium, leeches and the lancet are not necessarily attended with fatality, and why compare these effects with those of an agent, in the administration of which, neither intelligence nor discretion are safeguards? And if it be admitted that antimony, opium, leeches and the lancet are occasionally fatal *per se*, is it not a question, worthy, at least, of consideration, whether the physician is right in adding to the deadly agents of the materia medica? The agents of the materia medica, named by Dr.

Bigelow, have an extensive relation to disease, and are, confessedly, powerful means in the treatment of a vast variety of diseases. But nothing of this kind can be claimed for chloroform; the only good effect it produces is the induction of a state of the nervous system in which consciousness is lulled to sleep. And Dr. Bigelow has learned, probably, by this time, that the fatality of anesthetic agents is far beyond the comparative proportion he made in the sunny days of etherization.

We have already shown, by the admission of Dr. Simpson himself, that there was more acknowledged fatality in his statistics of puerperal anesthesia, than in the same number of cases where no agents of the kind were employed. It is certainly much more the duty of the physician to study how he can do the greatest amount of good to his patients, than to make experiments to ascertain how near he can come to killing a patient, without doing it. In employing a driver for a carriage we should certainly confide much more in one who would give a dangerous place as wide a berth as possible, rather than one who would drive so as barely to escape. And multitudes of the cases of chloroformizing very strongly resemble the cases referred to by Dr. James Johnson, in which heroic doses of medicine had been given. He said an examination of the statements left one in doubt whether they were great cures or great escapes.

Let us now consider some of, what may be called, the minor evils of anesthesia, reported by the friends of chloroform. We think they are sufficient, of themselves, to make men pause and consider well. It is now admitted by the warmest advocates of anesthesia, that its agents are comparatively useless in natural labors, but some still urge their use in preternatural cases. Dr. Ranking says: "It is abundantly evident, as it appears to us, that, judiciously administered, *excepting in a few cases of idiosyncrasy*, it [chloroform] is not only innocuous both to mother and child, but that the different stages of labor are passed through with a diminution of suffering, and also that a positive mechanical improvement in the physical condition of the parts implicated is brought about. It is a question whether its use is to be advised indiscriminately in those cases of natural labor in which the pains are comparatively slight; but we do not hesitate, taking the present aspect of the question to be the true one, to state, that in every case of natural labor, in which the suffering is inordinately great, or whenever operative interference is necessary, anesthesia *ought* to be induced; and we moreover consider that the accoucheur who, under such circumstances (no special contra-indications existing), neglects to avail himself of the inestimable benefits thus placed within his reach, neglects a large portion of the duties which are attached to his responsible office."

Dr. Ranking "excepts" a "few cases of idiosyncrasy," and if he had given us criteria, by which to determine, a priori, these cases of idiosyncrasy, he would have removed a formidable objection to the remainder of his remarks. The difficulty of resolving the question whether the anesthetic agent will produce an evanescent or a final insensibility, constitutes, in our judgment, almost an insurmountable objection to its use. It is a direful duty inflicted upon the feelings of a sympathetic mind, to stand by the bed-side of a delicate female and see her writhing in agony through the tedious hours of labor; but it is as dust in the balance, when compared with the feeling that we are looking upon the chloroformized corpse of one who confided her all to our keeping, and who was made a victim by that confidence. Medical art is widely astray from its province, when it places a patient in a condition of peril that art cannot relieve. Of the truth of this position there can be no doubt. If we had means by which to tell when it would be safe, and when perilous, to resort to anesthesia; if we could reason safely from one administration of its agents to another; or even if we could control their direful effects when they begin to show themselves, there would be weight in the urgent remarks of Dr. Ranking, but in the entire absence of all such safeguards, they are entitled to but little consideration.

Dr. Edward Murphy urges the following conclusions:

1st. "Chloroform does not interfere with the action of the uterus, unless given in large doses, which is unnecessary."

2nd. "It causes a greater relaxation in the passages and perineum. The mucous secretion from the vagina is also increased."

3d. "It subdues the nervous irritation caused by severe pain, and restores nervous energy."

4th. "It secures the patient perfect repose for some hours after delivery." There are cases where these few hours of "perfect repose" have extended into eternity.

5th. "*Its injurious effects, when an ordinary dose is given, seem to depend upon constitutional peculiarities*, or improper management."

In the first part of this fifth division, Dr. Murphy admits a fact, that, in our judgment counterbalances all the good results of his four preceding divisions. He admits that "injurious effects, from ordinary doses, seem to depend on constitutional peculiarities," and the ever-recurring objection springs up—we cannot determine the existence or non-existence of these "constitutional peculiarties" beforehand, and one fatal case would far outweigh years of successful practice. We cordially subscribe to the sentiment of Dr. Tyler Smith, published in the London Lancet. He says: "I do not envy the remorse which must follow the conviction, that by such practice the momentous arrangements of a dying hour have been entirely prevented."

We return to a consideration of Dr. Murphy's deductions, as they seem to cover the whole ground. He asserts that "chloroform does not interfere with the action of the uterus," but in this he is not borne out even by the advocates of anesthesia. Dr. Denham, in an able report on the use of chloroform, in the "Dublin Journal of Medical Science," says: "When a patient in labor is first brought under the influence of chloroform, it will generally be found that the pains are diminished in force, frequency and duration. After some time they generally regain much of what they had lost, and though apparently weaker, they yet seem to tell with equal or even better effect upon the progress of the labor. The muscles of animal life are first affected, *and if a complete state of anesthesia be induced, the action of the uterus is impaired.*"—Dr. Denham's cases fully substantiate the latter statement. In his third class of cases, "in which chloroform appeared to be not only useless, but, when persevered in, positively injurious," we meet with such details as the following:

"She was put under the influence of chloroform, but the pains soon became weak, and at the end of an hour ceased entirely; the chloroform was now left off, soon after which the pains increased to such a degree that we began to dread rupture of the uterus."

In another case, "the state of anesthesia was soon induced, and easily kept up. * * * In twenty minutes after she began to inhale she appeared to be almost in a state of coma—the pains gradually diminished, and at the end of two hours had almost entirely ceased. The chloroform was now discontinued, when the pains returned with considerable force."

In Dr. Denham's 12th case, he says: "Instrumental assistance, from the state of the parts, was out of the question, and I determined to try the effects of chloroform, to mitigate, if possible, her agonizing screams, which accompanied each pain. She was speedily under its influence, * * * and it decidedly put an end to her expressions of agony; but the labor pains nearly ceased, and for two hours and a half that I kept her in a state of anesthesia more or less profound the child never advanced."

Case 13th. "She was put under the influence of chloroform when in the second stage of labor, the pains at that time being regular and the child advancing; at the end of fifty-three minutes we were obliged to discontinue the chloroform, as the pains had almost entirely ceased. Soon after this they again returned, and the child was born in two hours."

Case 14th. "She was put under chloroform, when turning was effected with great ease; uterine action was completely put a stop to, and did not return after the feet were brought down. We were, therefore, obliged to discontinue the chloroform; the pains soon returned, and the labor was completed in a short time."

These are conclusive facts upon Dr. Murphy's first deduction.

We cannot acquit ourselves of a duty, towards this Report of Dr. Denham's, which we recognize and perform with pleasure, without commending it in the strongest terms to the attention of our readers. It is the most philosophical and practical monograph we have seen on the subject of chloroform in its application to midwifery. The closing remarks are conceived in a most commendable spirit, and expressed in beautiful language. Dr. Denham says: "We have yet much to learn about anesthetic agents. Should my example induce others to follow up the subject, I shall feel satisfied. The object of all interested in this question ought to be, and I am sure is, to benefit mankind. However much, therefore, we may differ upon this or any other subject, I trust we shall ever be found debating, not as the briar with the thistle, which can wound the deepest, but as the olive with the vine, which can bear the best fruit, and which most mitigate the sufferings of fallen humanity in the hour of nature's trial."

On the subject of Dr. Murphy's second deduction, the advocates of anesthesia are by no means agreed. They do not confirm the statement that "the passages and perineum are greatly relaxed" by chloroform. Dr. Simpson quotes one of these advocates as saying, that "this supposed effect, appears to him 'at least uncertain and accidental,' and that out of twenty-five cases in which he used it, he had two instances of rupture of the perineum,"* certainly not a very brilliant or conclusive proof of "greater relaxation." Another of Dr. Simpson's protogees, quoted by Dr. Montgomery, says: "that the influence of this drug, by accelerating the second stage of labor, especially towards its termination, by removing the resistance made by the muscles at the outlet of the pelvis, entails a risk of laceration of the perineum in certain cases, 'unless very great care is taken.'"

We find, then, that all the important and valuable deductions of Dr. Murphy are errors, the advocates of anesthesia being the judges in the case.

One other point, on the fact of the cessation of uterine pain, under the influence of chloroform, must not be forgotten, and that is the possibility of the occurrence of uterine hemorrhage in this state of things. All accoucheurs will admit that it may come on in these circumstances, and if it were to happen, during the cessation of the pain, the administrator of the chloroform would find he had more on his hands than he bargained for.

Of the effects of anesthetic agents upon the brain, we have much testimony, but can now refer to but a very small portion of it. M. Dubois says that in one of his

---

* Dr. Montgomery on Anesthetic agents.

patients, "the most intense premonitory signs of convulsion were induced; the congestion was so great that he almost expected the eyeballs to syringe forth blood." Dr. Tyler Smith says: "The morbid phenomena fairly attributable to ether, (a safer agent, be it remembered, than chloroform,) in cases which have recovered, have been nausea, sickness, stertorous breathing, pulmonary and cerebral congestion, convulsions, and protracted failure of the heart's action; a sad list, truly. These facts, and three deaths, at least, from the new agent, chloroform, besides many other serious results, will probably satisfy most practitioners that 'a meddlesome midwifery' is still a bad midwifery. In common with most teachers, I have long inculcated at Guy's Hospital, 'that unnecessary interference with the providentially arranged process of healthy labor is sure, sooner or later, to be followed by injurious and fatal consequences.'"

On the subject of the application of anesthetic agents in surgical operations, the clear-headed and admirable Professor Syme thus speaks: "In many operations it is of the utmost importance that the patient should retain a voluntary control over his movements, not only for assisting the operator by executing those that he may desire, but by abstaining from those which would be obstructive to the object in view. Thus I have known the little operation for fistula in ano not only impeded but prevented by the convulsive efforts induced through the use of ether. In all careful dissections, as those for hernia, and the removal of tumors from intricate connections of importance; I would, therefore, advise against inhalation. I lately disarticulated a clavicle from the sternum, for osteo-sarcoma, and dissected out some large deep-seated tumors of the neck, with results which, I believe, might not have proved so satisfactory if ether had been used. In operations affecting the nose and mouth, also, I should think it inexpedient to render the patient insensible; lest, from the want of voluntary effort, suffocation, or an approach to it, might arise from the entrance of blood into the air passages. Finally, I beg to warn against administering the ether to persons in a state of great weakness or exhaustion."

We have thus endeavored to present the subject of anesthesia as it stands at present. The heated zealots who first mounted the hobby endeavored to browbeat all opposition to the general use of anesthetic agents, and their immoderate boasting of invariable success reminded us forcibly of the mummeries of mesmerism. Some of the early publications on the effects of ether and chloroform, and in considering anesthesia they must be looked upon as one thing, read and sound very much like Dr. Elliotson's trumpetings in favor of Mesmerism. But experience and observation have materially modified that indiscriminate use of anesthetic agents which threatened at one time to inundate the whole practice of surgery, medicine proper, and midwifery. Men have been taught that for mere oblivion of feeling, the peril of anesthetics is too great, and those who administer them should always find some apology in the peril of the patient, without them. A practitioner might be justified in using these agents in a case of labor requiring instruments, when he could not be for using them in a case of natural labor.—We do not universally condemn the practice of giving chloroform, for in the use of the forceps, or crotchet, or in a difficult case of turning, in midwifery, the cautious use of the medicine may be beneficial. No amount of caution is sufficient to guard against the evil effects of chloroform in all cases, but there is much less danger in a prudent and cautious exhibition of these agents, than in a rash and indiscreet use of them. And we regard the perilous character of anesthesia as a boon of no ordinary moment in midwifery, for if it were otherwise, there would be a large increase of that meddlesome interference that could not fail to be baneful. If these anesthetic agents were innocent in their own results, they would be highly injurious in paving the way to an extended application of instruments in cases of labor, and that would be a serious evil. When Dr. Haighton, of London, was seventy years of age, he declared that he had delivered thirty thousand women, and amidst the multitudinous instances of deformed pelvis and depraved constitution to be found in the population of London, he said he had used instruments in but three cases, and he solemnly declared, that he believed his right arm should be taken off as the penalty for their use in at least two of these cases. "Experience," he said, "had satisfied him that if nature had been left to herself, she would have accomplished the labors without his assistance."

We rejoice to know that the use of anesthetic agents has been greatly modified, and that they are not now resorted to upon any and all occasions. The range of cases that demand their use has been materially diminished, and their application to that limited range, will lessen, rather than increase.

*Collodion.* The attention of the profession has been called to this new agent in surgery, and it is justly regarded as an important addition to the means of usefulness in the hands of the surgeon. It is one of the results of the increased attention of late to the uses of ether, and, as in the case of the solution of camphor in chloroform, shows that the progeny may improve upon the parent stock. As an excellent method of procuring adhesion between divided living parts, the solution of gun cotton in ether is a valuable discovery. Its use will grow upon the esteem of those who test its merits. As an adhesive article its value is very great. It is not influenced by heat or cold, or water, and it passes rapidly from a fluid state to a firm and persistent coating over a wound. In "cuts, burns, excoriations, and sore nipples," it has been highly commended, but, if we may judge from experience, not more so than it deserves.

Dr. Erasmus Wilson bears strong testimony to the value of collodion in diseases of the skin. He says he has used "it with advantage in chronic erythema of the face; intertrigo; chapped nipples and chapped hands; herpes labialis, preputialis, and herpes zoster; lichen agrias; lupus non exedens, and exedens; acne vulgaris; and several affections of the sebiparous organs. We are able to corroborate Dr. Wilson's strong testimony to the value of collodion in various diseases of the skin. In a troublesome and obstinate case of erysipelas, in which nearly the whole round of ordinary treatment was tried, without any benefit to the patient, the collodion worked like a charm. All other applications seemed to be injurious, but the disease commenced improving soon after the collodion was used, and it is now so near well, that we have no fear but that it will be thoroughly cured.

In ulceration of the os and cervix uteri, Dr. Mitchell, of Dublin, says the collodion is the best application he has ever used. After the ulcerated surface has been thoroughly dried, by repeated applications of lint, the collodion is applied with a camel's hair pencil, through a speculum. Repeated coatings are to be made, until a firm and adhesive varnish is attached to the ulcerated surface. At the end of forty-eight hours the dressing is to be renewed. In simple abrasions, Dr. Mitchell says this is sufficient, but where large granulations are present, it is necessary first to resort to nitrate of silver, or acid nitrate of mercury; after which the collodion may be used with advantage.

In the application of collodion to chronic ulcers, Dr. Startin, of the London Cutaneous Infirmary, gives the following directions: "Dry the ulcer well with bibulous paper; wash over its surface with a large soft brush, wetted with ether; dry a second time with the paper; apply, with a brush, the collodion in a circular manner, so as to cover the edges of the ulcer to a greater or less extent, as may be deemed necessary, and varnish over so much of the ulcer itself as to leave *a small central opening* for the escape of the discharges." "This expedient," says Dr. Startin, "reduces a large sore into a small one, and does not prevent any stimulus judged favorable to cicatrization being applied in the dry form, before the varnishing process is commenced."

In order to give the collodion a tint resembling the skin, Dr. Startin recommends the addition, to the collodion, of an ethereal solution of turmeric or saffron, and of red saunder's wood, or alkanet root, so as to produce the required tint. And to render the collodion opaque, more elastic, and to remove its great contractility, Dr. S. recommends the addition of linseed, cocoa nut, pure cod-liver, or lard oil, previously dissolved in ether, in the proportion of from half to a drachm of oil, to the ounce of collodion. The best materials should be used.

Dr. Comstock, of Wrentham, U. S., has applied collodion to a case of extensive laceration of the perineum with a success that he thinks never attended any former method of treatment. He says: "The dressings remained firmly attached, and solid, during the process of healing, notwithstanding they were for a time almost constantly covered by urine, mucus, and subject to being displaced by the motions of the patient."

In a recent number of the "Journal de Pharmacie et Chimie," we find a paper by M. Ilisch, of St. Petersburg, on the use of a combination of collodion and cantharides, for the purposes of vesication. The important point in his paper, is his method of preparing the compound, and we submit it to the attention of our readers.

Preparation—Take one pound of cantharides coarsely pulverized, with one pound of sulphuric ether, and three ounces of acetic ether, and mix them together. In this way we obtain a saturated solution of cantharides, and an animal matter of a green color. In two ounces of this liquid, dissolve twenty-five grains of the gun cotton.

This preparation may be kept without alteration in a close phial, and offers an excellent method of vesicating any part of the surface. The blistering agent cannot slip about, and vesicate parts that do not need it. And from a drachm and a half of this preparation we obtain as much effect as we could from half an ounce of the ordinary blistering ointment.

M. Livonius, of Neustrelitz, in the same Journal, gives a report of a series of experiments in making a collodion of remarkable adhesive power. He says that in making this improved collodion, he takes 200 parts of dry nitre, which he pulverizes finely, and 300 parts of strong sulphuric acid. The two substances are mixed in a porcelain mortar, and stirred with a glass tube. While stirring, add, as quickly as possible, 10 parts of pure cotton, very dry. After being in contact three minutes, during which it is continually stirred, the cotton is washed in distilled water, pressed and dried at a low temperature. But this does not make a complete solution, and M. Livonius says, that by observing the same proportions as above, mixing the dry nitre with English sulphuric acid, and leaving them in contact a space of five minutes, and then continuing the former operations, he obtained an article which dissolved with facility in sulphuric ether, and which makes a collodion of remarkable adhesive power. 5 grains of the cotton thus prepared, agitated in 110 grains of sulphuric ether, with the addition of 20 grains of alcohol, make a complete and transparent solution of the consistence of gum mucilage.

We cordially commend the collodion as a valuable and useful remedy in many cases, and as an important aid to other remedies, in various affections. Its application to its various uses, cannot fail to strike the attention of the intelligent practitioner.

